# Specific Abilities in the Workplace: More Important Than *g*?

**DOI:** 10.3390/jintelligence5020013

**Published:** 2017-04-12

**Authors:** Harrison J. Kell, Jonas W.B. Lang

**Affiliations:** 1 Academic to Career Research Center, Research & Development, Educational Testing Service, Princeton, NJ 08541, USA; 2 Department of Personnel Management, Work, and Organizational Psychology, Ghent University, Ghent 9000, Belgium

**Keywords:** intelligence, specific abilities, job performance, relative importance analysis, nested-factors model, specific aptitude theory, general mental ability, bi-factor model, hierarchical factor model, higher-order factor model

## Abstract

A frequently reported finding is that general mental ability (GMA) is the best single psychological predictor of job performance. Furthermore, specific abilities often add little incremental validity beyond GMA, suggesting that they are not useful for predicting job performance criteria once general intelligence is accounted for. We review these findings and their historical background, along with different approaches to studying the relative influence of *g* and narrower abilities. Then, we discuss several recent studies that used relative importance analysis to study this relative influence and that found that specific abilities are equally good, and sometimes better, predictors of work performance than GMA. We conclude by discussing the implications of these findings and sketching future areas for research.

## 1. Introduction

Differential psychologists have studied intelligence for over 100 years. The psychometric tests intended to measure intelligence are lauded as one of psychology’s greatest accomplishments [1,2]. The consistency and intensity of the attention devoted to intelligence is partly due to its power to predict many important practical outcomes [3,4]. One of these outcomes is job performance. Indeed, of all of differential psychology’s many constructs, general mental ability (GMA; *g*) is widely regarded as the best predictor of job performance [5]. Over the past 35 years, GMA’s priority has been established by primary studies, e.g., [6,7,8,9] and meta-analyses, e.g., [10,11,12,13]. Although few doubt that GMA is a powerful predictor of job performance, less consensus exists as to whether narrower, more content-aligned abilities are equally valuable predictors of workplace outcomes (Appendix A1). Despite strongly titled articles, e.g., “Predicting job performance: Not much more than *g*” [9] and repeated demonstrations that specific abilities add little incremental validity beyond GMA, e.g., [14], a recent survey [15,16] indicated that there is disagreement among both intelligence researchers and applied psychologists and that this a “critical area in need of additional research” (p. 685).

This paper reviews recent research on the topic of the relative roles of GMA and specific abilities in predicting workplace performance. We discuss the historical background of the debate about the primacy of general versus specific abilities and two alternative approaches to evaluating the relative influence of *g* and narrower abilities on work performance. We then review several recent studies from the industrial/organizational (I/O) literature that demonstrate the substantial power of specific abilities for predicting job performance. We conclude by discussing the implications of these studies for evaluating the situational specificity hypothesis and sketching several fruitful areas for future research. Our ultimate goal is to introduce intelligence researchers to some novel, recent findings in the applied psychology literature that are relevant to the ongoing cf. [15,16] debate about the relative influence of specific abilities and GMA on job performance.

## 2. Historical Backgroundd

Since the modern inception of cognitive ability testing, the emphasis has vacillated between generality and specificity [17,18]. Generality was initially dominant. Charles Spearman [19] famously postulated that a single entity he termed the “general factor” or “*g*” could largely explain the association between performance on disparate cognitive tests; he sometimes equated *g* with “mental energy” [20]. Spearman’s work was based on his observation that tests of cognitive abilities are typically positively correlated. In his psychometric analyses, Spearman typically extracted *g* and conceptualized specific abilities as the amount of variance that remains in each test after *g* is removed. Spearman believed that this approach was justified because he viewed *g* as the source of the shared variance among the tests. One year later, Alfred Binet and Theodore Simon developed the first practical measure of intelligence, the core of which Binet considered to be “judgment” [21]. In accordance with this outlook, the Binet-Simon test produced a single test score, which would eventually evolve into the intelligence quotient (“IQ”).

This emphasis on the generality of intelligence crossed the Atlantic when American practitioners and researchers learned of, and began to administer, Binet’s test. Henry Goddard produced the first English translation of the test in 1908, which was followed by many others [22]. Lewis Terman [23] produced a major revision of Binet’s test; the Stanford-Binet continued to generate a single score representing test-takers’ performance. Terman and Goddard were later members of the Committee on the Psychological Examination of Recruits, which was responsible for developing tests for evaluating potential recruits upon the entry of the United States into World War I [22]. The Committee produced the Army Alpha (for literate English speakers) and Army Beta tests (for illiterate or non-English speakers), which were administered to over 1.7 million men [24] and, too, produced single scores. The differences in Army Alpha scores were subsequently found to differentiate people across many practically important criteria, such as success in officers’ training camps, military rank, and occupation [25,26]. The emphasis on generality continued into the 1920s, with single scores being used to rank occupations in terms of their intellectual demands, e.g., [27] and classify children as being intellectually gifted, e.g., [28].

Interpretations of cognitive abilities that acknowledged a greater role for specificity had existed alongside more global conceptualizations since nearly the beginning of modern intelligence research, e.g., [29,30,31]. However, it was not until the late 1920s, with the publication of major works by Truman Kelley [32] and Edward Thorndike [33], that the field began to turn toward specific cognitive ability constructs in earnest [34]. Influenced by Thomson and Thorndike, Hull [35] proposed that the best way to predict outcomes such as job or training performance was to combine differentially weighted scores on tests of specific abilities (e.g., mechanical, quantitative, verbal), rather than using a single, global score (i.e., IQ). Over time this approach evolved into *specific aptitude theory*, which gave priority to specific abilities over general ability for predicting practical outcomes.

The tide definitively turned toward specific abilities in the U.S. with Thurstone’s [36] development of multiple factor analysis [37], which culminated in the creation of the Primary Mental Abilities taxonomy (PMA) [38]. PMA postulated seven uncorrelated specific abilities and, in doing so, denied the influence of GMA. The “discovery” of specific abilities quickly proliferated, with some postulating 40 to over 120 abilities by the 1970s [39]. The conceptual emphasis on specific abilities influenced the development of new, multi-attribute assessments over this period, such as the General Aptitude Test Battery (GATB), Army General Classification Test (administered to over 12 million World War II recruits [40]), Armed Services Vocational Aptitude Battery (ASVAB), and the Air Force Officer Qualifying Test [41,42]. In time the focus on specific abilities grew so intense that Ghiselli [43] (p. 83) could assert: “Whatever happened to Spearman’s G? Gone with the wind”.

GMA was not gone however, theoretically or practically. Although the work of Thurstone, Guilford, and others undeniably established the presence of specific abilities beyond *g*, scores on tests of these specific abilities were themselves correlated, implying the presence of a more fundamental—“higher order”—latent trait that accounted for differences among them. This higher-order construct was eventually acknowledged to be analogous to Spearman’s *g*, leading to the eventual theoretical reconciliation of general and specific approaches to cognitive abilities, e.g., [44,45]. Higher-order conceptualizations of cognitive abilities are currently the dominant approach [46], as represented by the Cattell-Horn-Carroll model [47,48], which features cognitive abilities at three levels of specificity, and the *g*-VPR model [49], which features four. Thus, although higher-order models acknowledge the presence of specific abilities, they also treat *g* as the construct that accounts for much of the variance in those abilities and, consequently, in whatever outcomes those narrower abilities are predictive of. Although these treatments differ somewhat in their details from, and are more sophisticated than, those of Spearman and Binet in their details, they ultimately share with their predecessors a prioritization of *g*, relative to specific abilities [17].

GMA has regained its practical importance, relative to specific abilities, as well. Not long before Ghiselli’s [43] dismissive statement, Jensen [50] began the project of resuscitating Spearman’s *g*; this task partially entailed demonstrating that specific abilities accounted for trivial incremental validity in workplace outcomes after accounting for GMA. This triviality was demonstrated repeatedly by Jensen [51,52] and others, e.g., [18,53,54], eventuating in a series of articles entitled “Not much more than *g*” by Ree and colleagues [9,55,56]. By the 2000s the idea that specific abilities are useful predictors of workplace outcomes after accounting for *g* appeared to have been all but wholly rejected [5,14,57,58,59]. Nonetheless, surveys that have explicitly asked for opinions on this matter reveal a lack of consensus on the relative role of specific abilities versus GMA for predicting external criteria [15,16].

## 3. GMA versus Specific Abilities and the Prediction of Job Performance

Specific aptitude theory has usually been evaluated using incremental validity analysis [60]. Scores for an external criterion (e.g., job performance) are regressed first on scores on *g*, with scores for specific abilities entered in the second step of a hierarchical regression. If the specific ability scores account for little to no incremental variance in the criterion beyond GMA, the specific aptitude theory is treated as being disconfirmed. In such an analysis, whatever variance shared between GMA and the criterion is attributed to the influence of GMA, because predictors entered into a hierarchical regression first have priority [61]. Furthermore, whatever variance narrower abilities share with the criterion that additionally overlaps with GMA is *also* attributed to GMA—because specific abilities themselves are conceptualized merely as “indicators” of GMA [14,58,59]. Only the variance shared between the criterion and narrower abilities that does *not* overlap with GMA is attributed to those narrower abilities. Such incremental validity analyses generally show that narrower abilities account for relatively little variance in criteria beyond GMA.

Intentionally or not, when researchers use incremental validity analysis to evaluate a specific aptitude theory, they invoke the assumption that an underlying latent trait (i.e., GMA) accounts for the variance shared across test scores. Lang et al. [60] have noted this view is theoretically close (although not identical) to Spearman’s original idea that all variance that tests in a battery share is attributable to the general factor, e.g., [62]. The idea that the variance shared by GMA and narrower cognitive abilities is attributable to GMA is also theoretically in line with higher-order conceptualizations of intelligence. In these conceptualizations, GMA is the underlying source of shared variance between the narrower cognitive abilities. Despite the prevalence of the practice, it is important for researchers and practitioners to realize that entering GMA first in incremental validity analysis is a choice—and not one they need to feel compelled to make.

If a researcher or practitioner (explicitly or implicitly) does not want to give GMA priority over specific abilities when evaluating their relative influences on external criteria, a different analytic strategy must be adopted, one that does not automatically attribute the variance shared between GMA and narrower abilities to GMA, as when GMA is entered first in the regression sequence. It might be assumed that scores on measures of narrower abilities and scores on a measure of GMA could simply be entered simultaneously in a regression and their respective standardized beta weights interpreted as indicators of the magnitude of their influence, but this is not the case: Researchers have long recognized that standardized beta weights do not sum up to *R*^2^ and do not adequately partition the variance shared among multiple correlated variables and the criterion [63,64,65,66]. Standardized regression coefficients are designed to measure the effects of predictors in specific combinations, and comparing two predictors using standardized regression coefficients contrasts the effects of the two predictors under different and very specific circumstances [64], such as comparing the effect of Predictor 1 given Predictor 2 (and possibly other variables) with the effect of Predictor 2 given Predictor 1 (and possibly other variables). The contribution a predictor makes alone (its direct effect) and in subsets of predictors (partial effects) is not considered [67].

The common goal in research on the importance of specific abilities and GMA is to rank-order predictors—but when predictors are correlated, betas do not provide a clear rank order [68]. An alternative, appropriate method for investigating the specific aptitude hypothesis is relative importance analysis [63]. This technique deals well with correlated predictors, which are ubiquitous in psychology [69] and particularly pervasive in intelligence research. Furthermore, relative importance analysis does not require a priori assumptions about variables’ priority and consequently does not automatically assign shared variance between *g* and narrower cognitive abilities to *g*, as incremental validity analysis does when *g* is entered in the first step of a hierarchical regression. (See Appendix A2 for a more detailed explanation of different analytic techniques for examining the relative influence of GMA and specific abilities.)

Given the pervasive usage of higher-order models [46], theoretically-minded researchers and practitioners may be concerned that using relative importance analysis to evaluate specific aptitude is inappropriate because it is not aligned with scientific approaches to studying cognitive abilities. This is not the case, however, as there are numerous models that do not make a priori assumptions about the presence of a single latent trait that accounts for abilities’ covariance. In the factor-analytic tradition, one such family of approaches is the nested-factor conceptualization, e.g., [70], where GMA and narrower abilities “compete” for shared variance. These conceptualizations date back to the work of Spearman’s collaborator Holzinger, e.g., [71], who invented the bifactor method. Holzinger and his colleagues used this method to derive a general factor by applying Spearman’s formula for extracting residuals across groups of tests that belonged to different specific factors. However, in Holzinger’s approach the variance that the specific tests shared with each other was assigned to the specific factors, so that GMA received relatively less variance than in the two-factor theory and itself depended on the extraction of the specific factors. Outside the factor-analytic tradition, models of intelligence like those of van der Maas et al. [72] and Thomson [73,74] are able to mathematically account for positive test intercorrelations without positing the presence of an underlying latent trait. Many of these models fit the data as well as the more commonly used higher-order approach [72,73,74,75] (Appendix A3).

We wish to be clear that neither incremental validity analysis nor relative importance analysis is more fundamentally “correct” in evaluating specific aptitude theory. Instead, we hold that they are equally appropriate, with each simply being a different way to tackle the same problem, and each being consistent with multiple prevailing structural models of intellectual abilities. If the desire is to give GMA priority over specific abilities—or, a measure of GMA is already present in a preexisting selection system—some use of hierarchical regression with GMA entered in the first step is recommended. If a preexisting selection system does not already include a measure of *g* or the desire is to not make assumptions about how variance is shared between GMA and narrower abilities, some variant of relative importance analysis is recommended. The studies we now review pursued this second strategy.

## 4. Recent Research Findings

**Figure 1:**
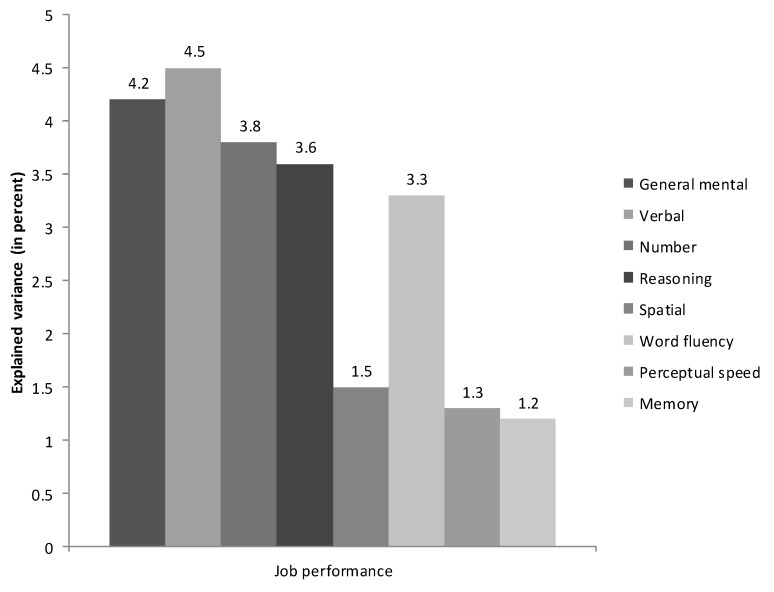
Relative weights analysis of the relative importance of general mental ability and Thurstone’s [38] Primary Mental Abilities in job performance in the meta-analytic study by Lang et al. [60].

**Figure 2:**
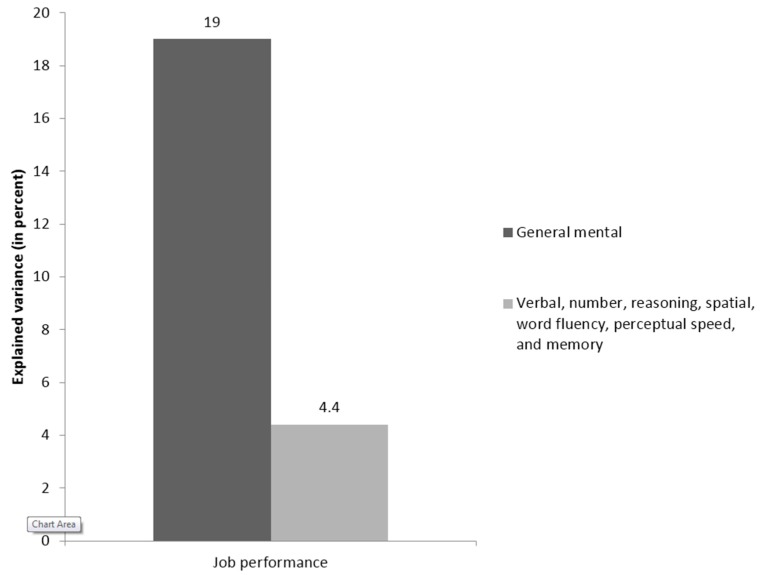
Incremental validity of Thurstone’s [38] Primary Mental Abilities over general mental ability in job performance in the meta-analytic study by Lang et al. [60].

Three recent studies used relative importance analysis to investigate the tenability of specific aptitude theory in the workplace. The results of the first study [60] are summarized in Figure 1 and Figure 2.

This study consisted of a meta-analytic integration of datasets examining the association between job performance and scores on the Wilde intelligence test. The Wilde intelligence test was originally developed by Klaus Wilde for the selection of civil servants in Germany and was based on Thurstone’s [38] PMA theory. The authors searched for prior research linking either the subtests with each other or to job performance; the result of this effort was a meta-analytic matrix with a harmonic mean of 2015 correlations linking all seven subtests from the Wilde and job performance.

Lang et al.’s [60] relative importance analyses revealed that *g* was not the most important predictor of job performance. Instead, the results suggested that verbal comprehension was more important than *g* and that number and reasoning ability were nearly as important as GMA. In contrast, when traditional incremental validity analyses were employed they produced results consistent with prior research disconfirming specific aptitude theory: *g* explained 19 percent of the variance in job performance, with the Primary Mental Abilities accounting for only an additional 4.4 percent of the variance in the criterion. The results of the meta-analytic study are consistent with the fact that the battery has frequently been used for administrative jobs in which verbal abilities are likely to be important. It is important to note that these analyses were conducted at the level of constructs, not measures: Relative importance analyses revealed the primacy of specific abilities after correcting for sampling error, unreliability of the predictors and criteria, and range restriction.

**Figure 3:**
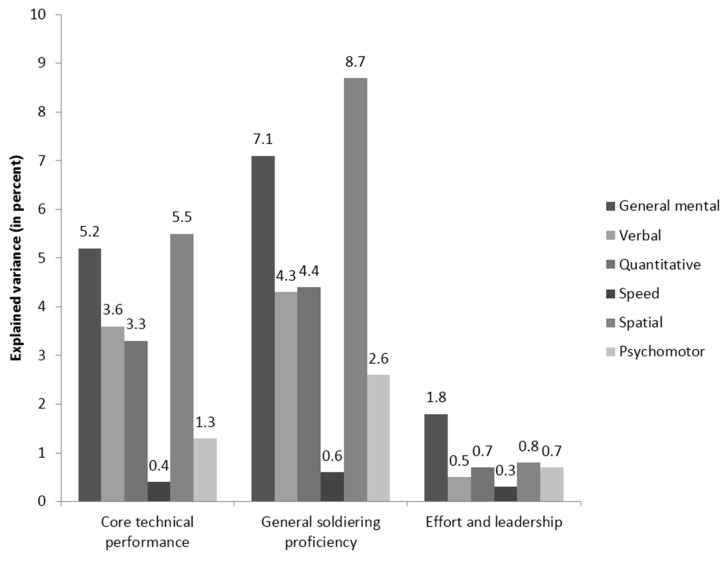
Relative weights analysis of the relative importance of general mental ability and five narrower cognitive abilities in three job performance criteria using the published correlation matrix from Project A [7]. Results were originally published in Lang and Bliese [76].

**Figure 4:**
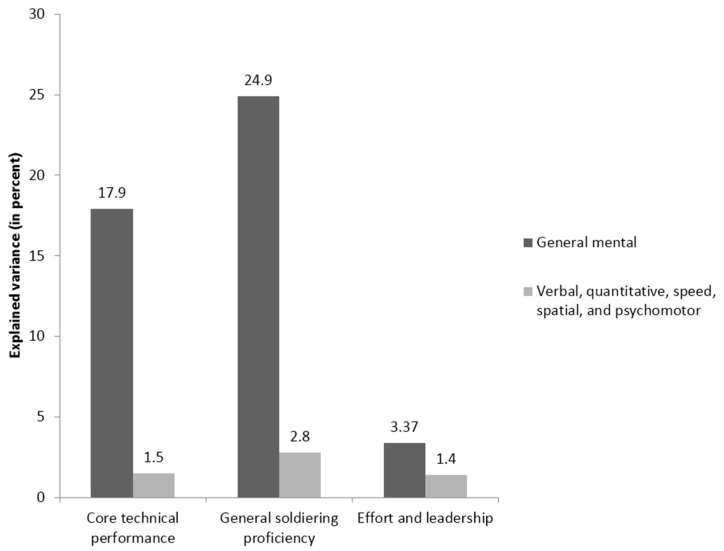
Incremental validity analysis of the incremental validity of five narrower cognitive abilities over general mental ability in three job performance criteria using the published correlation matrix from Project A [7]. Results were originally published in Lang and Bliese [76].

The second study [76] was a reanalysis of the Project A dataset [7]; its results are summarized graphically in Figure 3 and Figure 4.

Project A is a well-known large-scale validation effort by the U.S. Army that was designed to thoroughly investigate the criterion-related validity of a large number of cognitive and non-cognitive predictors in a sample of 4039 soldiers, each of whom performed one of nine jobs. The reanalysis focused on the relationship between cognitive tests and three major job performance criteria. When a traditional incremental validity was performed, *g* was the most important predictor among the cognitive abilities assessed, in alignment with the earlier Project A findings, e.g., [7]. Once more, however, when the relative importance analysis was employed, *g* was not the most important predictor. Instead, the results suggested that spatial abilities were more important than GMA for two of the three criteria (core technical proficiency and general soldiering performance). This finding is not surprising, given that most U.S. Army jobs include technical tasks of a mechanical nature and the fact that previous findings have frequently shown the critical importance of spatial abilities in technical jobs [77,78].

**Figure 5:**
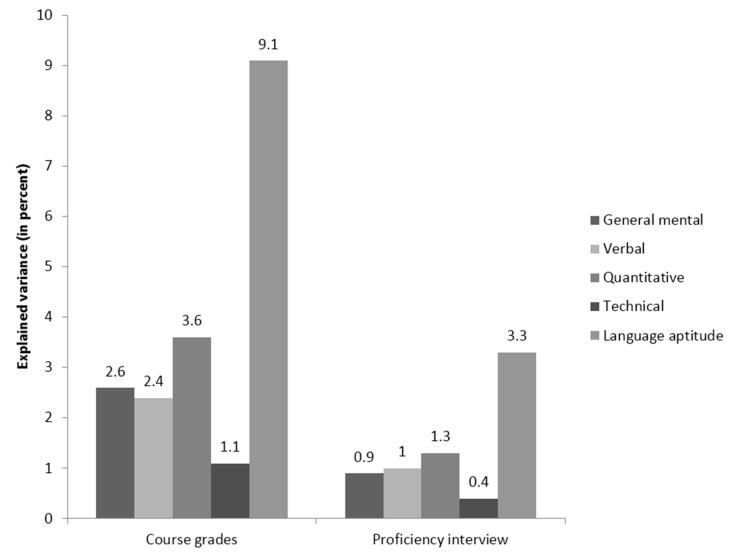
Relative weights analysis of the relative importance of general mental ability and four narrower cognitive abilities in two training success criteria in the Stanhope and Surface [79] study.

**Figure 6:**
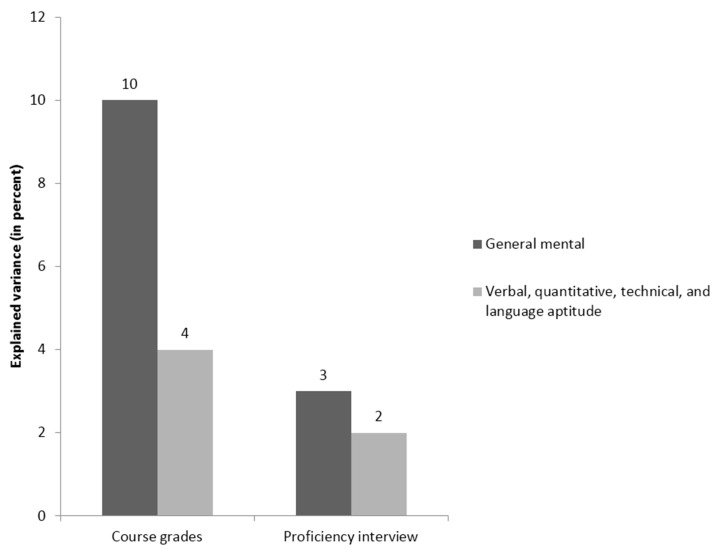
Incremental validity analysis of the incremental validity of four narrower cognitive abilities over general mental ability in two training success criteria in the Stanhope and Surface [79] study.

The third study [79] examined the success of military personnel that participated in a training for a foreign language, with the criteria being course grades and an oral proficiency interview; its results are summarized in Figure 5 and Figure 6.

Predictors included a GMA score, a verbal ability score, a quantitative ability score, and a technical ability score from the Armed Services Vocational Aptitude Battery (ASVAB). Also included was a language proficiency score from the Defense Language Aptitude Battery (DLAB). Stanhope and Surface’s [79] study is especially interesting because one of the predictors (DLAB) was particularly well-aligned with the criterion (language learning), while the others were not. In line with the results of the two previously described studies using the technique, the relative importance results suggested that the DLAB was a superior predictor of both criteria compared to GMA, along with the other predictors. Additionally, once more, the incremental validity analyses came to different conclusions. In these analyses, all narrower predictors only explained four percent additional variance in course grades, and two percent in the proficiency interview, after accounting for *g*. Furthermore, when the DLAB score was entered after the specific scores from the ASVAB, language proficiency did not explain additional variance in the criterion. On the basis of these incremental validity analyses, it could be argued that the DLAB adds little information beyond the ASVAB. Relative importance analyses came to the exact opposite conclusion: They indicated that language proficiency was the *most* important predictor of training success. This is particularly noteworthy given the content alignment of the DLAB with the criteria for foreign language training success, which suggests it could have substantive advantages over the ASVAB for giving feedback and explaining selection decisions. 

## 5. Implications for Situational Specificity

The results of the three studies reviewed appear to have implications for the situational specificity hypothesis (SSH) and they do—although not necessarily in an entirely straight-forward way. First, it is important to distinguish between two forms of SSH—“strong” and “weak”. The strong form is exemplified by the SSH definitions provided in the following quotes: “unknown influences within research settings” [80] (p. 257) and “unspecified subtle but important differences from job to job and setting to setting in what constitutes job performance, and that job analysts and other human observers are not proficient enough as information processors to detect these critical elusive differences” [81] (p. 402). The weak form of SSH is exemplified by Murphy’s [82] (p. 198) quote “If the correlation between the test scores and job performance truly depends on the job, organization, or situation, validity is said to be situationally specific” and the fact that Tett, Hundley, and Christiansen [83] (p. 14) define SSH as “increasingly specified conditions represented in a given aggregation”, and then list eight moderator classes (e.g., predictor constructs, general methods, criterion constructs) to account for when considering the SSH. The fact that two distinct hypotheses exist under the same label (the jingle fallacy [84]) may spring from the fact that defining what “situation” even means in the context of SSH has rarely been attempted [82]. The coexistence of two distinct forms of SSH may partially explain why it can be stated in pieces published two years apart that SSH has been “discredited” [85] (p. 30) and that “Support for the SSH is easily found in the literature” [86] (p. 910).

The strong SSH postulates a vague, unknown, unhypothesized moderator of validity across studies and Hunter and Schmidt [81] recommend rejecting it according to the “rule of thumb” that if 75 percent or more of the between-study variance in validity can be accounted for by artifacts, situational specificity is not present. The weak SSH merely postulates that there are moderators of validity whose presence can be hypothesized a priori, usually in accordance with some theory; to evaluate the weak SSH, studies in a meta-analysis merely need to be subgrouped according to the hypothesized moderator class(es) and the differences between them tested for [81]. 

The implications of using relative importance analysis to evaluate specific aptitude theory has immediate implications for evaluating the weak SSH, not the strong SSH. Lang et al.’s [60] results support the weak SSH. They found that GMA accounted for the most variance in performance in high complexity jobs but verbal comprehension accounted for the most variance in performance in low complexity jobs. The finding that the specific ability that accounted for the majority of the variance in the criterion differed across the three studies reviewed also *suggests* support of the weak SSH, although the data would have to be cumulated in a meta-analysis and subgroups formed to explicitly test it. (It is also important to note that of the three studies, only Lang et al. [60] corrected for artifacts.) Examination of these three studies reveals two viable, potential moderators: job type and criterion. Lang et al.’s [60] meta-analysis featured a wide variety of jobs and focused on overall job performance and found verbal ability was the most powerful predictor; Lang and Bliese [76] focused on military jobs, three distinct job performance criteria, and found spatial ability to be the best predictor; Stanhope and Surface [79] also used data gathered in a military context, but the criteria were course grades and interview performance after undergoing a foreign language training course, and they found scores on a language proficiency test (DLAB) to be the best predictor. Relative importance-based reanalysis of large datasets that contain information drawn from many different jobs may find that job type (or family) influences whether GMA or a specific ability is the best predictor of performance in that job. Indeed, Lang et al.’s [60] finding that GMA is the dominant predictor for highly complex jobs but verbal comprehension is the dominant predictor for low complexity jobs suggests that this may be the case.

In addition to focusing on different jobs, the three studies reviewed utilized different criterion constructs and measures. Across the three studies, the predictor that accounted for the most variance in performance was closely aligned with the criterion conceptually. These results not only suggest that the type of criterion is a plausible moderator of the relative importance of cognitive abilities for predicting performance, but also the criterion measure itself. For example, it is an empirical question if “overall performance” is measured by a single global rating made by a supervisor or if a weighted combination of supervisory ratings on specific job performance dimensions will influence the relative importance of abilities. Indeed, although there is a strong positive manifold among ratings provided by supervisors of various job performance dimensions [87], the intercorrelations are not unity, suggesting that different approaches to assigning performance ratings may influence which abilities are most influential in predicting them. When non-ratings based criteria are added (e.g., absenteeism, sales performance) the intercorrelation of criterion measures is lower than when ratings alone are used, e.g., [88,89,90], further implying that the ordering of abilities’ predictive validities may be moderated by both the criterion construct and how that construct is measured. Indeed, it has been openly speculated whether the consistent dominance of GMA over specific abilities would be maintained if the criteria beyond ratings of overall performance were more frequently employed [91]. 

It is important to be clear what evaluating weak SSH in terms of the relative importance of abilities means: It concerns the consistency of the ordering of the abilities that account for the most variance in the criterion across moderator classes. (Recall that weak SSH and validity generalization are not necessarily antagonistic—a construct may demonstrate substantial validity across all moderator classes but the magnitudes of the coefficients may still differ across those classes [82].) Lang et al.’s [60] results support the weak SSH because GMA accounted for the most criterion variance in high complexity jobs but verbal comprehension accounted for the most criterion variance in low complexity jobs. It is noteworthy, however, that in high complexity jobs verbal comprehension still accounted for 18 percent of the criterion variance—far from insubstantial. Thus, when evaluating the weak SSH in terms of relative importance it is critical not to ignore the absolute variance accounted for by each ability in each moderator class, in addition to the ordering of the abilities in each moderator class. It is conceivable that an ability may account for more criterion variance in a moderator class where it is not the dominant predictor than in a moderator class where it is; just because an ability is not the most important predictor does not imply it is unimportant.

## 6. Future Directions

The three studies we reviewed suggest there is potential for revitalizing specific aptitude theory by using relative importance analysis. It is important to emphasize that all three studies showed that avoiding the assumption that specific abilities are merely “indicators” of GMA can sometimes eliminate the common finding that GMA is vastly superior to specific abilities for predicting practical outcomes. However, these studies also showed that when the traditional approach of using incremental validity analysis is adopted, that the familiar finding of GMA explaining the majority of the variance in practical outcomes often follows. The three studies reviewed demonstrate two equally viable approaches for examining the relative usefulness of *g* and specific abilities for predicting real-world criteria.

Practically, an attractive characteristic of choosing between general or specific cognitive abilities based on relative importance analysis is the fact that it may give organizational and other decision makers the possibility of selecting among a set of nearly equally important predictors of the same outcome criteria while still remaining consistent with an underlying theory of cognitive abilities. Legal frameworks or face validity concerns (e.g., applicant reactions) may suggest that a particular predictor may be more desirable for an organization. For instance, an organization that seeks to select aviation engineers may decide to use spatial ability instead of GMA if both predictors are equally valid predictors of performance according to a relative importance analysis; in this particular context, spatial ability tests may possess greater face validity. Applicants may react more positively to spatial tasks than pedigrees, synonyms, and antonyms, because the content of the former is clearly related to the aerodynamic principles involved in airplane design while content of the latter tests is not. Indeed, researchers have suggested that relative importance analyses in general may be useful for establishing the most robust predictor of a criterion [92]. In line with this idea, one would expect that the specific abilities that are the most important predictors in a relative importance analysis may also robustly predict job performance in a particular type of job—but research must be conducted to empirically test this idea. 

In the previous section we outlined several classes of potential moderators that are worthy of further research: job type (or family), criterion construct, and criterion measure. To this we add the extent of the match between a predictor and criterion’s bandwidth cf. [93,94,95,96,97,98,99]. Given prior evidence that prediction tends to be maximized when the bandwidth of predictors and criteria is well-aligned, and that the general factor is by definition very broad, relative importance analyses would be expected to reveal that GMA is a better predictor of broad job performance criteria (e.g., “overall job performance”) and specific abilities are better predictors of narrower performance criteria (e.g., citizenship, counterproductive work behavior). Although this supposition is reasonable, it remains an empirical question that can only be answered with data—both in terms of its overall tenability and the appropriate level criteria that must be pitched to maximize the predictive validity of specific abilities as traditionally conceptualized and measured. For example, it is possible that even the narrower criterion dimension of “citizenship” is too broad to be well-aligned with specific abilities and should be further subdivided into personal support, organizational support, and conscientious initiative dimensions [98]. Future studies could also investigate differential weighting of the specific abilities and the narrower performance dimensions [99], bringing this line of research full circle and back to the proposal that was one of the seminal developments in specific aptitude theory [35].

Along these lines, relative importance-based analyses should be expanded to samples beyond those already examined. Of the three studies reviewed, Lang et al.’s study [60] was a meta-analysis of German studies, while Lang and Bliese [76], and Stanhope and Surface [79] were single studies conducted in the United States. Targeting large-scale international datasets, along with conducting meta-analytic reviews that estimate and correct for statistical artifacts (e.g., range restriction, sampling error), will be essential for establishing population-level relative importance estimates of GMA versus narrow abilities for work criteria, both within- and between-countries. These analyses could also be extended to other practically important variables, such as academic achievement and health outcomes.

## 7. Conclusions: The Resurgence of Specific Abilities?

We have reviewed three studies that demonstrate the practical value of studying specific cognitive abilities. These three studies are part of what appears to be a larger trend: The last decade or so has witnessed an apparent resurgence of interest in the predictive power of narrower cognitive abilities. Even using traditional regression techniques, recent studies have variously demonstrated the importance of narrower abilities above and beyond *g*. Using traditional regression techniques, Mount, Oh, and Burns [8] demonstrated the incremental validity of perceptual speed and accuracy over GMA for warehouse workers’ performance. Using ASVAB and GATB data, Wee, Newman, and Joseph [100] showed that differentially weighting specific abilities can maintain predictive validity and reduce adverse impact. More generally Krumm, Schmidt-Atzert, and Lipnevich [101] dedicated a special issue (partially inspired by Lang et al.’s [60] study) of *Journal of Personnel Psychology* to the role of specific abilities at work and Reeve, Scherbaum, and Goldstein [102] and Schneider and Newman [103] explicitly called for greater attention to specific abilities in human resource management and I/O psychology.

Specific abilities have also been the focus of concerted research in the realm of education and achievement testing, despite prior work being unsupportive of specific aptitude theory, e.g., [104,105,106,107]. Reeve [108] showed relations between specific abilities and knowledge scores in specific content areas using latent variable models. Studies by Coyle and colleagues, e.g., [109,110] have repeatedly demonstrated that standardized test scores, even after variance due to GMA is removed, predict college grades and that these residualized scores are correlated with specific abilities [111]. In a comment for this journal, Coyle [112] noted that one of the most important scientific issues in intelligence research is identifying constructs that possess validity after controlling for *g*.

Despite the growing emphasis on *g* in the 1980s, in the final edition of his *Essentials of Psychological Testing*, Cronbach [113] (p. 436) predicted: “Future personnel research will surely reinstate appreciation of multiple aptitudes.” The three studies we have reviewed, in concert with those just cited, suggest Cronbach may be proven right after all.

## Appendix A

### Appendix A1

We use the terms “specific abilities” and “narrower abilities” interchangeably throughout. These terms are intended to denote abilities that are clearly interpretable in terms of some broad content domain, such as Thurstone’s [38,44] Primary Mental Abilities, the abilities in the second stratum of Carroll’s [47] model, or the abilities in the second or third strata of Johnson and Bouchard’s [49] *g*-VPR model. This terminology does *not* refer to “extremely specific abilities” that may be relevant only to a single test or job.

### Appendix A2

The following provides a more technical explanation of the relationship among different models of cognitive abilities, various analytic methods, and the evaluation of specific aptitude theory.

Given *g* and a single narrower cognitive ability *n*, we can predict job performance in three different ways:

Scenario 1: Control *n* for *g*, then predict performance based on *g* plus the residuals of *n*. Here, *g* explains variance in *n and* job performance; variance in job performance is also explained by the remaining variance in *n* after controlling for *g*.

Scenario 2: Control *g* for *n*, then predict performance based on *n* plus the residuals of *g*. Here, *n* explains variance in *g and* job performance; variance in job performance is also explained by the remaining variance in *g* after controlling for *n*.

Scenario 3: Do not residualize *g* or *n* but instead use them both as predictors of job performance; variance in job performance is explained by variance in *g* and *n*.

Scenarios 1 and 2 are equivalent to a hierarchical regression analysis where *g* or *n* are entered first, yielding the squared semipartial correlation coefficient. According to Cohen and Cohen [61], the variance shares in this type of analysis depend on causal priority when a causal interpretation is desired. The predictor that is entered first has causal priority over the other predictor. This type of analysis can also be viewed from the perspective of practical predictor, when the research question entails studying whether an existing prediction approach with a predictor or a set of predictors can be improved by adding an additional predictor.

Scenario 3 is a standard regression analysis that yields beta coefficients, which provide insights into the effect of *g* on performance controlling for *n* or *n* on performance controlling for *g*. The beta coefficient is very closely related to the semipartial correlation coefficient, to the extent that the two are nearly identical in practice unless a beta coefficient becomes negative. Comparison of the formulas for the two metrics the reason for this close similarity:

Standardized regression coefficient

$$\beta_{Y1.2}=\frac{r_{Y1}-r_{Y2}r_{12}}{1-{r_{12}}^{2}}$$

Semipartial correlation

$$r_{Y\left(1.2\right)}=\frac{r_{Y1}-r_{Y2}r_{12}}{\sqrt{\left(1-{r_{12}}^{2}\right)}}$$

The two differ solely in that the standardized regression coefficient has no square root in its denominator while the semipartial correlation coefficient does, thus allowing the regression coefficient to exceed 1.0 in absolute value. Scenarios 1 and 2 yield semipartial correlation coefficients, while Scenario 3 yields beta coefficients—which the above equations indicate are very closely related. The analyses in all three scenarios provide insight into the degree to which a predictor explains variance the criterion after variance due to another predictor has been removed.

Scenario 1 is aligned with the “classic” conception of cognitive abilities, where *g* accounts for the intercorrelation of specific abilities. Scenario 2 is aligned with a conception of abilities that treats specific abilities as accounting for variance in *g*. In this conception, *g* might be considered an emergent property of specific abilities, as has been suggested by Johnson and Deary [114] and van Der Maas et al. [72]. In this scenario *g* could also be considered a “summary” of more specific abilities, e.g., [115], an intervening variable rather than a construct [116]. Analyses aligned with Scenario 2 seem to be rare, although it has been suggested that it is just as appropriate to ask “What does *g* add beyond specific abilities?” as the more familiar “What do specific abilities add beyond *g*?”, e.g., [117,118]. Scenario 3 is aligned with an agnostic approach to the relationship between GMA and specific abilities but is analytically problematic due to the limitations of beta weights when predictors are highly correlated [65]. A possible solution is to first perform a hierarchical regression with *g* entered first and *n* entered second, perform a second hierarchical regression with *n* entered first and *g* entered second, then derive the overall *R*^2^ for each variable by averaging the *R*^2^ associated with it in one regression with the Δ*R*^2^ associated with it in the other regression. (For example, the overall *R*^2^ for *g* would be the mean of the values of its *R^2^* from the first regression and its Δ*R^2^* from the second regression.) This approach is, essentially, dominance analysis [64], which is very similar to relative importance analysis [119], which we now discuss.

The current paper is concerned with studies focused on a fourth scenario, one concerned with relative importance, which can be defined as “the contribution a variable makes to the prediction of a criterion variable by itself and in combination with other predictor variables” [119] (p. 2). In Scenario 4 a major goal is to compare (i.e., rank order) predictors in terms of their ability to predict an outcome. Another goal of relative importance analysis is to distribute the *R^2^* amongst given predictors without making inferences about priority, which are implicitly invoked in Scenarios 1 and 2. Modern relative importance measures have the desirable property that they can be summed up to *R*^2^, while squared beta coefficients cannot be; the same is true for squared semipartial correlations. Earlier research, like the classic Cohen and Cohen [61], explored what they referred to as “commonality analysis” (variance jointly explained by two correlated predictors). However, in standard regression the communality can be negative, so Cohen and Cohen [61] argue that this type of analysis has limited usefulness; relative importance analysis addresses this issue of the standard regression procedure.

In sum, standard regression and relative importance analysis are different approaches for partitioning explained variance and for estimating and conceptualizing the contribution of correlated predictors. Each of these approaches is designed to answer a particular type of research question. If the research question is focused on examining the respective influence of GMA versus specific abilities for predicting external criteria and GMA is treated as the latent trait underlying specific abilities, the question becomes one of comparing the unique contribution of specific abilities beyond GMA to the contribution of GMA itself. This research question can be answered through hierarchical regression by examining the *R*^2^ for GMA alone and comparing it to the incremental *R^2^* contributed by specific abilities. Usually, *R*^2^ for GMA is found to be many times greater than the *R^2^* for specific abilities. If the research question is focused on examining the respective influence of GMA versus specific abilities for predicting external criteria and an agnostic attitude is taken toward the relationship between GMA and narrower abilities, it becomes a case of relative importance. Relative importance analysis can answer this question by providing a rank-ordering of GMA and specific abilities in terms of their relationship with the external criterion of interest.

We thank Paul de Boeck for his delineation of Scenarios 1–3.

### Appendix A3

Those with purely applied concerns will also find relative importance analysis useful, as it represents an additional, practical tool for studying the relative influence of specific and general abilities in the workplace. Some practitioners have indicated tests of specific abilities are preferable to tests of GMA because they often engender more positive applicant reactions, are more legally defensible, and demonstrate smaller group differences, e.g., [120], providing empirical grounding for their usefulness that is critical in applied settings.
